# Uncovering obsessive-compulsive disorder risk genes in a pediatric cohort by high-resolution analysis of copy number variation

**DOI:** 10.1186/s11689-016-9170-9

**Published:** 2016-10-18

**Authors:** Matthew J. Gazzellone, Mehdi Zarrei, Christie L. Burton, Susan Walker, Mohammed Uddin, S. M. Shaheen, Julie Coste, Rageen Rajendram, Reva J. Schachter, Marlena Colasanto, Gregory L. Hanna, David R. Rosenberg, Noam Soreni, Kate D. Fitzgerald, Christian R. Marshall, Janet A. Buchanan, Daniele Merico, Paul D. Arnold, Stephen W. Scherer

**Affiliations:** 1 The Centre for Applied Genomics and Program in Genetics and Genome Biology, The Hospital for Sick Children, Toronto, ON Canada; 2 Department of Psychiatry and Program in Genetics and Genome Biology, The Hospital for Sick Children, Toronto, ON Canada; 3 Mathison Centre for Mental Health Research and Education and Hotchkiss Brain Institute, Cumming School of Medicine, University of Calgary, Calgary, AB Canada; 4 Faculty of Medicine, University of Toronto, Toronto, ON Canada; 5 Department of Psychiatry, University of Michigan Medical School, Ann Arbor, MI USA; 6 Department of Psychiatry and Behavioral Neurosciences, Wayne State University, Detroit, MI USA; 7 The Children’s Hospital of Michigan, Detroit, MI USA; 8 Department of Psychiatry and Behavioural Neurosciences, Faculty of Health Sciences, McMaster University, St. Joseph’s Healthcare, Hamilton, ON Canada; 9 Departments of Psychiatry and Medical Genetics, Cumming School of Medicine, University of Calgary, Calgary, AB Canada; 10 Department of Molecular Genetics and McLaughlin Centre, University of Toronto, Toronto, ON Canada

**Keywords:** Obsessive-compulsive disorder, Copy number variation, Pediatrics, Whole-exome sequencing

## Abstract

**Background:**

Obsessive-compulsive disorder (OCD) is a heterogeneous neuropsychiatric condition, thought to have a significant genetic component. When onset occurs in childhood, affected individuals generally exhibit different characteristics from adult-onset OCD, including higher prevalence in males and increased heritability. Since neuropsychiatric conditions are associated with copy number variations (CNVs), we considered their potential role in the etiology of OCD.

**Methods:**

We genotyped 307 unrelated pediatric probands with idiopathic OCD (including 174 that were part of complete parent-child trios) and compared their genotypes with those of 3861 population controls, to identify rare CNVs (<0.5 % frequency) of at least 15 kb in size that might contribute to OCD.

**Results:**

We uncovered de novo CNVs in 4/174 probands (2.3 %). Our case cohort was enriched for CNVs in genes that encode targets of the fragile X mental retardation protein (nominal *p* = 1.85 × 10^−03^; FDR=0.09), similar to previous findings in autism and schizophrenia. These results also identified deletions or duplications of exons in genes involved in neuronal migration (*ASTN2*), synapse formation (*NLGN1* and *PTPRD*), and postsynaptic scaffolding (*DLGAP1* and *DLGAP2*), which may be relevant to the pathogenesis of OCD. Four cases had CNVs involving known genomic disorder loci (1q21.1-21.2, 15q11.2-q13.1, 16p13.11, and 17p12). Further, we identified *BTBD9* as a candidate gene for OCD. We also sequenced exomes of ten “CNV positive” trios and identified in one an additional plausibly relevant mutation: a 13 bp exonic deletion in *DRD4*.

**Conclusions:**

Our findings suggest that rare CNVs may contribute to the etiology of OCD.

**Electronic supplementary material:**

The online version of this article (doi:10.1186/s11689-016-9170-9) contains supplementary material, which is available to authorized users.

## Background

Obsessive-compulsive disorder (OCD) is a heterogeneous neuropsychiatric condition characterized by persistent intrusive unwanted thoughts (obsessions) and repetitive behaviours (compulsions) [1]. It is among the most common neuropsychiatric disorders, with a lifetime prevalence of ~2 % [2]. Males and females are affected in similar numbers (1:1 sex ratio), although males typically experience symptoms earlier than do females [1, 2]. First-degree relatives of individuals with OCD are significantly more likely to have OCD than are relatives of controls [1]. Classification of individuals into more homogeneous subtypes has been used to increase the likelihood of identifying causal factors [3]. One such approach takes advantage of the bimodal distribution of age-of-onset, with one peak in childhood and another in mid-adulthood [4]. The 30–50 % of individuals who first experience symptoms before age 18 may represent a distinct subtype of OCD [5]. On average, these individuals have worse symptom severity scores, respond less to pharmacological interventions, and have higher rates of tic disorders and attention deficit hyperactivity disorder (ADHD) compared with individuals with adult onset OCD [6–8]. Most important, the duration of OCD symptoms in childhood is a significant predictor of how persistent these behaviours will be in adulthood, stressing the significance of early diagnosis and intervention [5, 6].

Higher heritability estimates in childhood-onset OCD relative to adult-onset OCD emphasize a more substantial role for genetic factors in these individuals, though the identification of specific genetic susceptibility variants requires additional investigation [1]. Studies of genetic linkage in large pedigrees and analyses of candidate genes have identified potentially contributory loci, but replication of these findings has been inconsistent [1]. The contribution of common variants to the disorder was explored via two genome-wide association studies (GWAS) [9, 10], and these studies only identified genes of suggestive relevance, possibly due to insufficient power. Altogether, findings from GWAS and candidate gene studies suggest that OCD is complex in origin, possibly involving perturbations in many genes, with suggestive etiological evidence for *DLGAP1*, *BTBD3*, *SLC1A1*, *FAIM2*, and *PTPRD* [1].

Gene dosage imbalances caused by rare copy number variations (CNVs) have been identified as plausible contributory factors in other neuropsychiatric conditions, particularly those that are de novo in nature [11–13]. Higher levels of de novo copy number variation are noted in individuals with a range of neuropsychiatric conditions compared to the general population and are typically a focal point of CNV studies of disease [14–16]. OCD traits often co-occur with such conditions, including in 30–40 % of individuals with autism spectrum disorder (ASD), [17] 7–17 % with schizophrenia, [18] and 11–21 % with bipolar disorder [19]. One study so far has examined the genome-wide contribution of rare CNVs to OCD [20]. It identified an association trend between deletions at 16p13.11 and OCD. The relatively large size cut-off used (500 kb) excluded the investigation of smaller CNVs.

To identify potentially contributory genetic factors in OCD, we conducted a CNV screen using high-resolution microarrays, which facilitated CNV calling down to 15 kb. We also performed exome sequencing of selected families, in search of additional contributory factors. Finally, we assessed the cohort with respect to its overall burden of mutation and enrichment of variants in functional gene sets.

## Methods

### Subjects and controls

Participants were recruited from four academic child psychiatry sites: The Hospital for Sick Children, McMaster University, University of Michigan, and Wayne State University. Subjects were enrolled via clinics (site clinics, other mental health providers and primary care physicians), the internet (e.g., www.umhealthresearch.org at University of Michigan), hospital and community bulletin boards, and paid and public service advertisements in local media.

All enrolled individuals (164 females and 143 males) had symptoms first identified before age 18 (mean, 7.9 ± 3.5 years). Respective institutional ethics review boards approved all procedures. Informed consent was provided by capable adolescents. For younger children, parents or other legal guardians provided written informed consent, and the children gave verbal assent prior to participating in the study. Criteria for diagnosis are in the Additional file 1.

Our unrelated population control data were from three cohorts: Cooperative Health Research in the Region Augsburg (KORA) [21], the collaborative genetic study of nicotine dependence (COGEND), [22] and the Ontario Population Genomics Platform (OPGP) [23]. The same quality control procedures and CNV calling algorithms applied to our subjects had been applied to these controls.

### Detection of rare copy number variants

We genotyped all 174 trios and 58 additional unrelated probands using the CytoScan HD array, and 75 unrelated probands using the OMNI 2.5 M array. We employed multiple algorithms to call CNVs from the CytoScan HD and OMNI 2.5 M microarray data. We defined a stringent set of variants wherein each variant was called by at least two algorithms (Additional file 2: Table S1) [24]. We defined the ancestry and relatedness of the samples using PLINK [25] following methods previously described in other studies (see Additional file 1) [26, 27]. This information was used to exclude related probands or controls and detect any sample mismatches.

To define rare CNVs, we first computed frequency using a pooled set of stringent CNVs from cases and controls, matching for ancestry, platform, and sex (Additional file 1: Figure S1). We then removed those CNVs present at >0.5 % frequency, using the 50 % reciprocal overlap criteria [11, 28]. Next, we required that CNVs overlap a region that is at least 75 % copy number stable according to the CNV map of the human genome [28]. Finally, we used a cut-off of 15 kb and 10 probes for any remaining CNVs, yielding a comprehensive and high-quality list of rare CNVs from our case and control cohorts. We first ascertained CNVs from all samples, including individuals of non-European or mixed ancestry and utilized the entire control set. We then restricted our analysis to cases of European ancestry (for the subsequent gene set analysis). Those CNVs listed in Table 1 are not present in population controls unless stated otherwise in the table. We validated de novo events and possible risk variants in proband and parental samples (when available) using a secondary confirmation method (a SYBR-green-based real-time quantitative PCR (qPCR) or a TaqMan real-time PCR assay) [27, 29]. Primer sequences are available upon request from SWS. Twenty-five of 28 CNVs of interest were validated (including all in Table 1 and 4/5 that were tagged as being de novo). Suspected mosaicism in Case D was confirmed using digital droplet qPCR (Additional file 1: Figures S2 and S3). All coordinates refer to human genome assembly build GRCh37, hg19.

**Table 1:** Copy number variants of potential clinical significance in OCD subjects

Case	Sample ID	Sex	Onset (year)	CNV type	Location^a^	Size (kb)	Genes	CNV source	Exome seq
*De novo*									
A	8961143	F	6	dup	Xp22.31	305	*VCX3B, KAL1*	*de novo*	✓
B	896993	M	8	del	4p16.3	165	*ADRA2C*	*de novo*	
				dup	3p26.3	1735	*CNTN6, CNTN4*	pat	
C	1254 (188613)	M	3	del	3p12.2	182	–	*de novo*	✓
				del	3q26.31	142	*NLGN1*	mat	
				del	7q36.2	115	*DPP6*	mat	
				dup	7q36.3	67	*PTPRN2* ^b^	pat	
D	896713	F	10	dup (mosaic)	10q11.22- q11.21	239	*ZFAND4, FAM21C, MARCH8*	*de novo*	nsf ^c^
				dup	10q24.1	81	*SLIT1* ^b^	mat	
Overlap with curated DECIPHER syndromes^d^									
E	HAM493	F	8	dup	15q11.2-q13.1	4918	13 genes	n/a	
F	0625-4262-1	M	8	dup	1q21.1-21.2	1799	15 genes	mat	✓
G	1648	F	12	del	17p12	1404	8 genes	mat	✓
				dup	5p11-p12 ^e^	1065	*HCN1* ^b^	pat	
H	896573	M	9	dup	16p13.11	783	8 genes	mat	nsf
Genes implicated in other neurodevelopmental disorders									
I	1298	M	5	dup	Xp22.31	338	*NLGN4X*	mat	✓
J	896673_a	M	12	del	9q33.1	173	*ASTN2, TRIM32*	n/a	
K	7542	M	12	del	9q33.1	163	*ASTN2, TRIM32* ^b^	n/a	
L	0625-3695-3	M	12	dup	12q24.33	682	*TMEM132D*	mat	✓
M	1688	F	3	del	18q22.1	2126	*CDH19, CDH7*	mat	
*FMRP* target genes									
N	HAM193	M	12	dup	9p24.1	1489	*PTPRD, C9orf123*	n/a	
O	896553	F	6	del	8p23.3	16	*DLGAP2*	pat	✓
P	1213	M	4	dup	18p11.31	62	*DLGAP1*	pat	✓
Overlap *BTBD9*									
Q	0625-3790-3	F	8	del	6p21.2	107	*BTBD9*	pat	
R	0625-7518-3	F	7	dup	6p21.2	469	*GLO1, DNAH8, BTBD9*	pat	

Abbreviations: *n/a* parental sample not available, *nsf* no significant findings, *FMRP* fragile X mental retardation protein

^a^Coordinates can be found in Additional file 2: Table S1

^b^One CNV of the same type with similar breakpoints was also identified in our population controls

^c^ *nsf* indicates that exome sequencing of these probands identified no *de novo* or neurologically relevant loss-of-function mutations

^d^DECIPHER list of expert-curated microdeletion and microduplication syndromes involved in developmental disorders

^e^Found in the same patient, but not itself associated with DECIPHER syndromes

### Burden of CNVs in cases and controls

We analysed the burden of all CNVs, and of deletions and duplications independently. We also looked at CNV burden in the following size ranges: >15 kb (all), 15–100, 100–500, >500 kb, and >1 Mb. A binomial logistic regression analysis determined whether the differences in gene rates (the average number of genes intersected by CNVs per subject) between cases and controls were the result of a biological mechanism or statistical chance. We corrected for the number of CNVs and the total length of CNVs in the subject in our model.

### Gene set enrichment analysis

We tested gene set enrichment using the rare variants detected for individuals of European ancestry (Additional file 1: Figure S4). This analysis focused on events impacting protein-coding exons, and considered deletions and duplications separately. The analysis consists of a case-control burden test with correction for the different platforms and also for global CNV burden modelled as total CNV gene count by subject (i.e., penalization for large CNVs that may drive results in an unspecific way). The gene sets tested were comprised of genes associated with neurological functions (Additional file 3: Table S2) [11, 30]. Gene set burden enrichment was performed using a logistic regression analysis deviance test corrected for gene count and global burden (Additional file 1). The genotyping platform used for CNV calling was modelled as a covariate. We undertook the analysis twice; first without exclusion criteria, and second, by excluding neurodevelopmental risk loci previously described [20, 31, 32]. This provided an opportunity to see whether any enrichment signal was being driven by loci already recognized. For final results, we utilized 15 % as the Benjamini-Hochberg false discovery rate significance threshold.

### Exome sequencing

For samples with notable CNV findings (Table 1), we selected those 10 families for which adequate sample material was available from the proband and both parents for additional exome sequencing. This was conducted using the Illumina HiSeq2500, following exome capture with Agilent SureSelect Human All Exon V5 target enrichment kit. We aligned sequences to the hg19 reference using Burrows-Wheeler Aligner (BWA) v0.5.9, and used the Genome Analysis Toolkit (GATK) v1.1–28 to detect single nucleotide and small insertion and deletion variants. Rare variants were defined as those with a frequency of <0.01 in 1000 genomes, National Heart, Lung, and Blood Institute (NHLBI) Exome Variant Server and Exome Aggregation Consortium (ExAc datasets; http://exac.broadinstitute.org/). A Residual Variation Intolerance Score (RVIS) [33] and Probability of Loss of Function (LoF) Intolerance score (pLI) [34] were retrieved for each variant discovered. We confirmed the presence of all variants in Table 2 in proband and parental samples using Sanger sequencing [35].

**Table 2:** All *de novo* or neurologically relevant loss-of-function mutations from exome sequencing of selected families

Case	Sample ID	Position	Gene	Isoform	Change	Effect	Source	RVIS (%)^a^	pLI^b^
A	8961143	chr4:69,403,498-69,403,498	*UGT2B17*	NM_001077	p.A480T	Missense	*de novo*	96.5	0.01
G	1648	chr18:3,102,478	*MYOM1*	NM_003803	p.G1190A	Missense	*de novo*	81.7	0.00
		chrX:114,347,884-114,347,887	*LRCH2*	NM_020871	p.C730fs	Frameshift del	*de novo*	53.2	0.92
J	896553	chr14:24,677,345	*TSSK4*	NM_001184739	p.T337M	Missense	*de novo*	77.7	0.00
		chr14:74,947,404	*NPC2*	NM_006432	c.548 + 1C > T	Splice site	mat	71.1	0.01
K	1213	chr9:2,824,808	*KIAA0020*	NM_014878	p.I348N	Missense	*de novo*	78.9	0.00
R	0625-3695-3	chr9:107,266,546	*OR13F1*	NM_001004485	p.M1I	Missense	*de novo*	95.9	0.00
			*USP54*	NM_152586	p.R1395X	Nonsense	pat	91.4	0.00
C	1254	chrX:148,048,382-148,048,393	*AFF2*	NM_002025	p.992_996del	Deletion	mat	7.2	1.00
		chr11:637,537-637,549	*DRD4*	NM_000797	p.A78fs	Frameshift del	mat	N/A	0.00
		chr3:129,155,547	*MBD4*	NM_003925	p.E314fs	Frameshift ins	mat	83.4	0.00
F	0625-4262-1	chr5:145,895,394	*GPR151*	NM_194251	p.R95X	Nonsense	mat	78.7	0.00
O	1298	chr19:11,325,007	*DOCK6*	NM_020812	p.Q1428X	Nonsense	mat	9.4	0.00

The above variants were validated in proband and parental samples using Sanger sequencing

^a^Residual Variation Intolerance Score

^b^Probability of Loss-of-Function Intolerance

## Results

### Rare copy number variations

To explore the contribution of rare CNVs to OCD, we used a comprehensive CNV calling procedure with experimental validation of relevant variants. CNVs from 307 probands passed quality control, including 174 for whom DNA from both parents was also genotyped. Of these 307 probands, 259 were of European ancestry. Data from 1773 European controls were treated as those from the cases.

We uncovered 729 rare CNVs from these European cases (Additional file 2: Table S1), and 5182 from the European controls. This represented a mean of 2.81 and 2.92 rare CNVs per individual, respectively. We considered the number of genes intersected by CNVs in cases compared with controls (Additional file 1: Table S3), but found no statistically significant difference in this respect when looking at all CNVs, or at deletions and duplications independently. We also looked for differences in a particular CNV size range but found no greater global burden for rare CNVs in cases compared with controls (Additional file 1: Table S3).

We screened 174 parent-child trios of all ancestries for rare de novo CNVs. In four of these probands (2.3 %) (Table 1, cases A–D), we uncovered a single de novo CNV (sometimes in addition to inherited variants).

Various genomic syndromes have been observed in neurodevelopmental disorders including autism, schizophrenia, and epilepsy [13]. We sought to identify in our cohort rare CNVs that have been noted in some of these syndromes using the DECIPHER database containing expert-curated microdeletions and microduplications involved in developmental disorders [36]. We also compared our results with a list of OCD and ASD risk genes [1, 11, 32, 37]. We identified four individuals (Table 1, cases E–H), each with a CNV whose breakpoints were compatible with those in DECIPHER. We identified other CNVs in genes with possible relevance to OCD (Table 1, cases I–M), in gene targets of the enriched gene sets, particularly the fragile X mental retardation protein (FMRP) (cases N–P), and in a new plausible candidate, *BTBD9* (cases Q–R).

### Exome sequencing

In 10 of the trios where we discovered a potentially relevant CNV (i.e., de novo, or involving gene(s) associated with neurological function), we followed our CNV analysis with exome sequencing of the proband and parents. We focused our analysis on any de novo mutations or any predicted loss-of-function mutations in genes considered to play a role in neurological function. From these families, we identified six de novo variants and seven rare inherited loss-of-function mutations in genes with a neurological role (Table 2). We subsequently obtained RVIS and pLI scores for each of the genes to show how able they are to tolerate functional genetic variation and loss-of-function mutations, respectively. Only one of the genes identified from exome sequencing, *AFF2*, had an RVIS score in the tenth percentile (among the most intolerant genes) and a pLI score of above 0.9 (a score considered to be suggestive of pathogenic LoF mutation) [34].

### Gene set enrichment

Our initial analysis sought to identify gene set enrichment, in cases compared with ethnically matched controls, from CNVs that impact a gene’s coding sequence (Table 3; Additional file 1: Table S4 and Additional file 4: Table S5). We found such enrichment for CNVs in FMRP target genes (nominal *p* = 1.85 × 10^−03^), both when we included or excluded known neurodevelopmental risk loci from the analysis. When such loci were included, cases were 1.75 times more likely to have one FMRP target impacted by a CNV than were controls, and 4.38 times more likely to have two FMRP targets impacted (Additional file 4: Table S5). Much of the signal seemed to be driven by duplications (nominal *p* = 0.014), though the limited sample size and false discovery rate (FDR) of 36 % cautions against over-interpretation. When considering all loci and a more relaxed FDR threshold (0.275) [38], cases were enriched with CNVs for genes involved in nervous system development (human neural function or pathway, nervous system development (GO)) (nominal *p* = 0.019) and the broader group encompassing genes involved in neurological function (human neural function or pathway, union, inclusive (GO, KEGG, NCI, Reactome)) (nominal *p* = 0.020). Similar results were generated when removing CNVs at known neuropsychiatric disease loci.

**Table 3:** Summary of enriched gene sets

Gene set	Size of gene set	With known loci^a^		Without known loci	
		Nominal *p* value	False discovery rate (FDR)	Nominal *p* value	FDR
*FMR1* targets^b^	840	0.00185	0.09	0.00312	0.16
Nervous system development^c^	1,874	0.0186	0.26	0.0633	0.46
Union inclusive^d^	2,874	0.0205	0.26	0.0385	0.35

^a^Loci implicated in neurodevelopmental disorders

^b^“Human neural selected components, FMR1 targets” in Additional file 3: Table S2; deletions and duplications

^c^“Human neural function or pathway, nervous system development (GO)” in Additional file 3: Table S2; deletions and duplications

^d^“Human neural function or pathway, union, inclusive (GO, KEGG, NCI, Reactome)” in Additional file 3: Table S2; deletions and duplications

### Notable case findings

#### De novo copy number variants (Cases A–D)

Case A has a de novo duplication intersecting *VCX3B* and *KAL1* on chromosome X. *KAL1* is involved in cell adhesion, neurite outgrowth, and axon guidance. *VCX3B* is expressed only in male germ cells. Her mother was diagnosed with an eating disorder.

We identified a de novo 165 kb deletion encompassing *ADRA2C* in Case B. *ADRA2C* regulates neurotransmitter release and modulates GABA release from the striatum. B also carries a large 1.7 Mb duplication impacting *CNTN6* and part of *CNTN4*, inherited from his father who has learning difficulties. CNVs involving each have been noted in autism cases [11, 16].

Case C had onset of OCD at age 3 years, and also has Tourette syndrome and ADHD. Parental phenotypes were not provided. Multiple CNVs included a de novo deletion in a non-genic region, maternally-inherited deletions of *NLGN1* and *DPP6*, and a *PTPRN2* duplication also seen in his father. Exome sequencing also revealed three maternally-inherited variants: a 13 bp frameshift deletion of *DRD4* (a dopamine receptor gene), a 4-amino-acid in-frame deletion of *AFF2* (also known as *FMR2)* and a single-base insertion in *MBD4*. *NLGN1* encodes a protein involved in forming excitatory synapses and maintaining synaptic plasticity [39]. Exonic CNVs of *NLGN1* and *DPP6* have been identified in individuals with autism [11, 40]. Duplications of *PTPRN2* has been implicated in ADHD [12]. Rare mutations in *AFF2* have been noted in ASD [41]. *DRD4* has been previously associated with OCD [42].

Case D is mosaic for a large de novo duplication overlapping *ZFAND4*, *FAM21C*, and *MARCH8*. None of these genes has been previously implicated in neuropsychiatric disorders. She also carries a rare exonic duplication in *SLIT1*, inherited from her mother who was diagnosed with depression. *SLIT1* is thought to play a role in axonal navigation and neuron projection.

#### CNVs known to be associated with curated DECIPHER syndromes

In female case E, we found a large 15q11-q13 duplication. About 1 in 500 children referred for genetic testing for developmental delay or autism has a CNV at this locus [43], and duplications of this region are among the most common genomic rearrangements seen in ASD probands, being present in 1–3 % of these individuals [44]. This locus includes the GABA_A_ receptor subunit gene cluster, disturbances of which are proposed to increase the risk of developing anxiety disorders [45]. The duplication of this gene cluster may be an important contributor to the observed OCD phenotype of this individual.

From his unaffected mother, case F inherited a large duplication overlapping the 1q21.1 locus, and he shares the duplication with his male dizygotic twin who does not have an OCD diagnosis. Duplications of this locus are variably associated with both child- and adult-onset neuropsychiatric conditions and congenital abnormalities [46]. They have also been observed in some individuals with ASD [47].

Case G has OCD with generalized anxiety disorder, social anxiety disorder, and specific phobia. We discovered a large maternally-inherited deletion at 17p12—a locus associated with hereditary liability to pressure palsies (HNPP)—in this individual. Deletions of this locus usually manifest in patients as peripheral nerve problems [48], though this patient has no such symptoms. This deletion has been noted in asymptomatic individuals and may not be relevant to the OCD phenotype of this individual [48]. She also carries a paternally-inherited duplication impacting the first five exons of *HCN1*, a gene encoding ion channels that facilitate synaptic integration and plasticity. Phenotypic information was not available for either parent.

We identified a maternally-inherited duplication overlapping the 16p13.11 recurrent microduplication locus in case H. This locus is associated with neurocognitive disorders, and is implicated in individuals with autism [11] and some with OCD [20], though deletions (not duplications) were the significant finding in the latter.

#### CNVs in genes implicated in other neurodevelopmental disorders

In addition to OCD, case I suffers from major depressive disorder, social anxiety disorder, and a panic disorder. We found a duplication of the first non-coding exon of *NLGN4X*, inherited from his mother (who was not examined phenotypically). Functional studies of *NLGN4X* have shown that the gene plays important roles with respect to neuronal development and neurite formation [49]. Elsewhere, brothers with Tourette syndrome—one with autism as well, and the other with ADHD—had a three-exon deletion of *NLGN4X* [50]. Interestingly, their mother who also carried the deletion had anxiety and depression, suggesting that variants of *NLGN4X* might predispose to a range of neuropsychiatric phenotypes.

*ASTN2*, a gene that plays an important role in neuronal migration, was found deleted in two males. Case J was previously reported by our group in an individual with OCD and social anxiety [29]. Case K, the other individual with an *ASTN2* deletion, has depression, ADHD, and Tourette syndrome. Both variants overlap the “critical region” at the 3’ end of the gene identified in a previous study [29].

In case L, we identified a duplication of *TMEM132D*, a gene implicated in panic disorder, and thought to contribute to anxiety phenotypes [51].

Case M has OCD with trichotillomania, and a deletion involving *CDH7* and *CDH19*. Her parents were not interviewed. Both genes encode members of the cadherin family—proteins that play an important role in brain development.

#### CNVs involving FMRP target genes

In case N, we found a 1.5 Mb duplication involving *PTPRD*, a gene that is highly expressed at the presynaptic terminal and regulates glutamatergic synapse development, and is a target of FMRP. This variant completely duplicates five of the six main isoforms, suggesting that increased dosage of the gene product may contribute to the disorder phenotype. In a large GWAS for OCD, a single nucleotide polymorphism (SNP) near this gene was also the marker with the smallest *p* value [10].

Case O inherited from her unaffected father a single exon deletion from *DLGAP2*—another FMRP target. This is the first study to identify a CNV in *DLGAP2* in an individual with OCD. However, previously, two SNPs in *DLGAP2* were nominally associated with the orbitofrontal cortex white matter volume in children with OCD (although this association was not significant after correction for multiple comparison) [52].

The duplication impacting *DLGAP1* in case P involves an exon that is considered to be “brain critical” [53]. This child also has Tourette syndrome, separation anxiety disorder, and oppositional defiant disorder. Though he inherited this variant from his unaffected father, his younger sister who carries the same variant was also diagnosed with OCD at age 4, and has social anxiety disorder, specific phobia, and transient tic disorder. Their mother possesses some features of OCD-like behaviour, but does not meet the criteria for diagnosis. This may suggest that other maternally inherited genetic factors also contribute to the phenotypes of the child.

#### CNVs impacting *BTBD9*

We identified two individuals with CNVs overlapping *BTBD9*. Case Q has a 107 kb exonic deletion inherited from her father (for whom phenotypic details were unavailable). Case R has OCD and Tourette syndrome, and inherited a larger duplication that overlaps the 5’ end of the gene. She inherited the duplication from her father who has seasonal affective disorder. This being the first time CNVs of *BTBD9* have been identified in individuals with OCD, we expanded our analysis by also inspecting our unpublished microarray, exome, and whole-genome sequencing data in autism cases for CNVs or loss-of-function mutations in this gene. We found a previously unpublished male ASD proband with a paternally-inherited single-base-pair deletion, predicted to cause a frameshift and premature truncation of the BTBD9 protein. This individual also displayed obsessive compulsive tendencies. Though his father had no evidence of OCD (only writing difficulties), the child’s paternal grandfather had a host of psychiatric issues including depression, poor social skills, substance abuse, OCD, and Tourette syndrome. DNA was not available from the grandfather to test for segregation of the variant.

## Discussion

We found de novo CNVs in 2.3 % of OCD cases—a rate lower than typically seen in other neurodevelopmental disorders such as schizophrenia (4.5 %) [14], bipolar disorder (4.3 %), [14] cerebral palsy (7.0 %) [26], or autism (4.7 %) [11], but slightly higher than for ADHD (1.7 %) [12]. The de novo rate in population controls from microarray is closer to 0.9–1.4 % [14, 54]. Although there is some variation between the different microarray platforms used, all are considered to be high-resolution arrays and all focus on CNVs in comparable size ranges to those we examined. As a result, a general comparison can be attempted with the caveat that this is a single study, and larger sample numbers for all disorders would even better highlight any differences that might exist. In addition, disparities in the selection of variants for validation and sample source might play a minor role in the differences observed. That said, there are also important biological reasons that could explain the differences inde novo rates in different disorders.

Individuals with autism, schizophrenia, and bipolar disorder have reduced reproductive rates [55] making it less likely that they pass on a newly acquired mutation; although reduced fecundity is also noted in OCD, it is only slightly lower than that of individuals without a neuropsychiatric condition [56]. Therefore, inherited genetic variation may play a more substantial role than de novo events in the underlying genetic architecture for OCD. We identified rare inherited CNVs at positions congruent with curated DECIPHER syndromic loci, and overlapping genes associated with other neurodevelopmental disorders, FMRP targets, or *BTBD9*. In 10/174 (5.7 %) of our trios, we identified an inherited genetic lesion that we propose might have contributed to the patient’s OCD phenotype. Four additional probands had possibly pathogenic copy number changes of unknown inheritance. In all, we identified candidate variants of potential significance to OCD in 18/307 cases (5.9 %).

Enrichment of CNVs in certain gene sets can highlight specific pathways or functional categories that contribute to OCD risk. In OCD cases compared with controls, we identified significant enrichment of rare CNVs intersecting genes involved in neurological function, particularly targets of FMRP (Table 3). Three CNVs overlapping FMRP targets—*PTPRD, DLGAP1* and *DLGAP2*—were of particular interest in this cohort. Two SNPs near *DLGAP1* showed the highest associations in a previous GWAS of OCD [9]. A marker near *PTPRD* exhibited the highest association with OCD in the second GWAS [10], and the locus has also been implicated in OCD by linkage studies [57, 58]. The enrichment in these targets is of particular relevance, given FMRP’s high expression in neurons and its role as a regulatory protein that alters the translation of proteins involved in synaptic function [59], making it essential for a range of cognitive processes. This concurs with studies of ASD and schizophrenia that suggest CNVs impacting FMRP targets increase risk for neuropsychiatric conditions [11, 60]. However, in contrast to the larger role for FMRP deletions in these disorders, duplications are potentially more relevant for OCD (statistically significant in our study, albeit with a high FDR). This may reflect an alternate mechanism underlying some of the relationship between genotype and phenotype.

Studies of OCD and of other neuropsychiatric conditions invoke a genetic model whereby the atypical development or maintenance of synaptic connections contributes to a neuropsychiatric phenotype [1, 61]. As a result, many of the same candidate genes are emerging across a range of neuropsychiatric disorders. Here, we have once again uncovered *ASTN2* variants in individuals with a neuropsychiatric condition. A previous study [29] that has shown CNVs of this gene often are observed in ADHD, and one individual in our study has this diagnosis in addition to OCD. We also identified a potential new OCD risk gene, *BTBD9*, which encodes a protein with a BTB/POZ domain. Proteins containing this domain play a role in synaptic plasticity and neurotransmission and alter dendritic branching.

## Conclusions

One particularly interesting area of further research would be to apply complementary microarray, exome sequencing, or whole-genome sequencing approaches [41, 62]. In autism, findings from exome sequencing and from CNVs are largely non-overlapping [63]. With this precedent, here, we undertook exome sequencing in 10 trios in our study, one of which revealed a plausibly relevant mutation (*DRD4*). This pilot study demonstrates the importance of using multiple approaches to investigate the genetic contribution to OCD.

We identified a number of CNVs that overlap risk loci for other neurodevelopmental conditions. As in McGrath et al. [20], we found no increased global CNV burden in cases compared with controls. Although we did not find 16p13.11 deletions in our study (the main finding in McGrath et al. [20]), we did identify a duplication at this locus. Most of our variants of interest were smaller than their 500 kb cut-off, stressing the importance of examining variation at all size ranges in OCD.

There are important caveats that must be accounted for when interpreting our study. The first is that our statistical power is constrained by our small sample size. Because of this, most of the rare variants that were identified in our cases only occurred once. As a result, we were required to rely to a greater extent on previously published work and functional studies to prioritize genes of interest. In addition, the small sample size resulted in higher false discovery rates than what we would wish, necessitating follow-up studies in larger cohorts of OCD patients. Unfortunately, in many of our families, we also lacked extensive phenotypic details in some patients and even more frequently, in parents, preventing more comprehensive genotype and phenotype correlation. That said, our study presents new avenues for further investigation and highlights some important candidate genes. Most importantly, we reaffirm the extensive genetic heterogeneity in neuropsychiatric conditions, while demonstrating overlap between genes uncovered here with those of other neurodevelopmental conditions.

## Additional files

Additional file 1: Supplementary Information. (DOCX 292 kb)
Additional file 2: Table S1 (XLSX 63 kb)
Additional file 3: Table S2 (XLSX 11 kb)
Additional file 4: Table S5 (XLSX 1313 kb)

## Abbreviations

ADHD: Attention deficit hyperactivity disorder
ASD: Autism spectrum disorder
CNV: Copy number variation
COGEND: Collaborative genetic study of nicotine dependence
DECIPHER: Database of Genomic Variation and Phenotype in Humans Using Ensembl Resources
FDR: False discovery rate
FMRP: Fragile X mental retardation protein
GATK: Genome Analysis Toolkit
GWAS: Genome-wide association studies
HNPP: Hereditary liability to pressure palsies
KORA: Cooperative health research in the Region of Augsburg
NHLBI: National Heart, Lung, and Blood Institute
OCD: Obsessive-compulsive disorder
OPGP: Ontario population genomics platform
qPCR: Quantitative polymerase chain reaction
SNP: Single nucleotide polymorphism
