# Supplementary material for: Uncovering obsessive-compulsive disorder risk genes in a pediatric cohort by high-resolution analysis of copy number variation
Source: J Neurodev Disord. 2016 Oct 18;8:36. doi: 10.1186/s11689-016-9170-9 (PMC5070001; doi:10.1186/s11689-016-9170-9)
Supplement: Additional file 1: — Supplementary Information. (DOCX 292 kb) [file 11689_2016_9170_MOESM1_ESM.docx]

**Supplementary Information for:**

**Uncovering obsessive-compulsive disorder risk genes in a pediatrics cohort by high-resolution analysis of copy number variation**

Matthew J. Gazzellone^1*^, Mehdi Zarrei^1*^, Christie L. Burton^2^, Susan Walker^1^, Mohammed Uddin^1^, S-M. Shaheen^2,3^, Julie Coste^2^**,** Rageen Rajendram^2,4^**,** Reva J. Schachter^2^, Marlena Colasanto^2^, Gregory L. Hanna^5^, David R. Rosenberg^6,7^, Noam Soreni^8^, Kate D. Fitzgerald^5^, Christian R. Marshall^1^, Janet A. Buchanan^1^, Daniele Merico^1^, Paul D. Arnold^2,3,9,10**^, Stephen W. Scherer^1,11**^

^1^The Centre for Applied Genomics and Program in Genetics and Genome Biology, The Hospital for Sick Children, Toronto, Ontario, Canada

^2^Department of Psychiatry and Program in Genetics and Genome Biology, The Hospital for Sick Children, Toronto, Ontario, Canada

^3^Mathison Centre for Mental Health Research and Education and Hotchkiss Brain Institute, Cumming School of Medicine, University of Calgary, Calgary, Alberta, Canada

^4^Faculty of Medicine, University of Toronto, Toronto, Ontario, Canada

^5^Department of Psychiatry, University of Michigan Medical School, Ann Arbor, MI, United States

^6^Department of Psychiatry and Behavioral Neurosciences, Wayne State University, Detroit, MI, United States

^7^The Children's Hospital of Michigan, Detroit, MI, United States

^8^Department of Psychiatry and Behavioural Neurosciences, Faculty of Health Sciences, McMaster University, St. Joseph's Healthcare, Hamilton, Ontario, Canada

^9^Department of Psychiatry and Institute of Medical Science, Toronto, Ontario, Canada

^10^Departments of Psychiatry and Medical Genetics, Cumming School of Medicine, University of Calgary, Calgary, Alberta, Canada

^11^Department of Molecular Genetics and McLaughlin Centre, University of Toronto, Toronto, Ontario, Canada

*co-first authors, with equal contribution to the study

**co-senior corresponding authors

**Supplementary Methods**

**Subjects**

At all sites, participants and their parents were interviewed with the Schedule for Schizophrenia and Affective Disorders for School-Aged Children — Present and Lifetime Version.[[1](#_ENREF_1)] In addition, the Schedule for Obsessive-Compulsive and Other Behavioral Syndromes [[2](#_ENREF_2)] was used at the University of Michigan and Wayne State sites. We assessed specific symptoms and current severity of OCD in the participants using the Children’s Yale-Brown Obsessive Compulsive Disorder Scale.[[3](#_ENREF_3)] The site clinical investigator — a child and adolescent psychiatrist — made lifetime and current axis 1 diagnoses using all sources of information according to DSM-IV criteria.

**Algorithms**

We used custom scripts written in R for the CNV analyses of this paper.[[4](#_ENREF_4)] Gene set burden enrichment was tested as implemented by cnvGSA (<https://www.bioconductor.org/packages/release/bioc/html/cnvGSA.html>).

**CNV detection**

For samples genotyped on the CytoScan HD, the following quality control process was utilized. First, all samples were required to have a MAPD (Median Absolute Pairwise Difference) of less than 0.25, a SNP QC above 15, and a Waviness SD score below 0.12. As in our previous studies,[[5](#_ENREF_5), [6](#_ENREF_6)] CNV calling was completed using the Affymetrix Chromosome Analysis Suite (ChAS) (Affymetrix Inc., USA), iPattern,[[7](#_ENREF_7)] Nexus,[[8](#_ENREF_8)] and Partek [[9](#_ENREF_9)] CNV calling algorithms. For autosomes, all CNV calls were required to have been made using at least two algorithms, with at least one being ChAS or iPattern. For calls on the X chromosome, only iPattern and ChAS were used and calls were required to have been made by both. When detecting CNVs from samples genotyped on the OMNI 2.5M microarray, a slightly different but comparable method was utilized. First, all samples were required to have a call rate exceeding 0.95. CNVs were then detected using iPattern,[[7](#_ENREF_7)] PennCNV,[[10](#_ENREF_10)] CNVPartition,[[7](#_ENREF_7)] and QuantiSNP.[[11](#_ENREF_11)] CNV calls on the autosomes required detection by at least two algorithms, with one being either PennCNV or iPattern. Calls from the X chromosome were only made with PennCNV and iPattern with both detecting a variant for it to be tagged as stringent. After calling, we removed any sample for which the total number of calls exceeded three times the standard deviation plus the mean number of calls for all of the samples.

**Ancestry detection**

To infer ancestry of the 232 samples typed on Cytoscan HD and 75 samples typed on HumanOmni2.5-Quad, we used HapMap3 samples typed on Genome-Wide Human SNP Array 6.0 (Affymetrix Inc., USA) as the reference set. The SNPs common to the three platforms were extracted, with filtering based on missingness and minimum allele frequency using PLINK.[[12](#_ENREF_12)] LD-based pruning of the autosomal SNPs with parameters 50 (window size), 5 (step), 0.5 (r^2^ threshold) yielded 83,396 SNPs. The top two principal components were plotted using scripts included in the package, to determine population structure. Samples were colour coded, and HapMap samples labeled according to the population of origin. The third iteration removed outliers. The results of this iteration are plotted in Supplementary Figure S4. Comparison with HapMap-CEU data suggested that no further iterations were required, and samples were tagged based on these results.

**Computing frequency using a pooled strategy and defining rare variants**

To define rare CNVs in samples from individuals of European ancestry, we pooled stringent CNV calls from the 63 European cases genotyped on the OMNI 2.5M with those from 2,563 European population controls tested on the same array. Similarly, calls from 196 European cases genotyped on the CytoScan HD were combined with those from 820 European population controls assayed on that array. We also combined calls from European cases genotyped on either platform with calls from 953 randomly-selected population controls tested on the OMNI 2.5M and 820 from the CytoScan HD array. Samples totalled 2,032, with half from each array and the sex ratio balanced. For cases including non-European samples, we compared with our entire cohort of population controls (2,988 KORA and COGEND and 873 OPGP). We then removed those CNVs present at >0.5% frequency in each such cohort, using the 50% reciprocal overlap criteria. [[13](#_ENREF_13), [14](#_ENREF_14)] We then applied additional filters to define final rare variants for our analyses (see main text).

**Gene sets**

For gene sets tested for enrichment, see Supplementary Table S2. The genes in each gene set have been previously published.[[15](#_ENREF_15)]

**Gene set enrichment**

We also considered whether the type of CNV had any effect on the enrichment findings, by examining whether the CNV encompassed an entire gene, or was contained within a gene. Such a finding might indicate a role for gene dosage or domain-specific variations as a general mechanism for increased risk.[[16](#_ENREF_16)] No gene set was significant when considering CNVs that completely encompassed a transcript. However, we identified enrichment of CNVs contained within the transcript for five gene sets, including our neurological function set (human neural function or pathway, union, inclusive (GO, KEGG,NCI, Reactome)), human neural function or pathway, nervous signal transmission (GO), human neural selected components, post-synaptic density, human neural selected components, ARC complex (activity-regulated cytoskeleton-associated), and human neural function or pathway, synaptic components (Supplementary Table S5). These findings further illustrate the importance of synaptic genes in the context of neuropsychiatric disorders.

**Supplementary Table S3: Analysis of global burden**

| **type** | **group size** | **number of rare genic CNVs** | **genes intersected by rare CNVs** | **baseline gene rate (cases)** | **baseline gene rate (controls)** | **case-control gene ratio** | **nominal p-value** |
| --- | --- | --- | --- | --- | --- | --- | --- |
| **all** | all | 2,222 | 2,804 | 2.38 | 2.36 | 1.01 | 0.88956 |
| **deletions** | all | 796 | 932 | 0.59 | 0.68 | 0.87 | 0.57191 |
| **duplications** | all | 1,426 | 2,076 | 1.79 | 1.68 | 1.07 | 0.58062 |
| **all** | 15-100kb | 1,301 | 1,266 | 0.85 | 0.96 | 0.89 | 0.23007 |
|  | 100-500kb | 756 | 1,208 | 0.90 | 0.92 | 0.97 | 0.86179 |
|  | >500kb | 165 | 749 | 0.63 | 0.49 | 1.30 | 0.57863 |
|  | >1Mb | 44 | 391 | 0.24 | 0.26 | 0.95 | 0.46671 |
| **deletions** | 15-100kb | 575 | 515 | 0.38 | 0.37 | 1.02 | 0.74824 |
|  | 100-500kb | 192 | 267 | 0.16 | 0.19 | 0.84 | 0.60236 |
|  | >500kb | 29 | 197 | 0.05 | 0.12 | 0.43 | 0.75714 |
|  | >1Mb | 10 | 142 | 0.05 | 0.08 | 0.57 | 0.75400 |
| **duplications** | 15-100kb | 726 | 801 | 0.47 | 0.59 | 0.81 | 0.22254 |
|  | 100-500kb | 564 | 978 | 0.73 | 0.73 | 1.01 | 0.66295 |
|  | >500kb | 136 | 562 | 0.59 | 0.37 | 1.58 | 0.22327 |
|  | >1Mb | 34 | 253 | 0.20 | 0.18 | 1.12 | 0.47497 |

**Supplementary Table S4: Enriched gene sets when considering CNV type**

| **gene set*** | **size of gene set** | **with known loci** | | **without known loci** | |
| --- | --- | --- | --- | --- | --- |
|  |  | **nominal p-value** | **FDR** | **nominal p-value** | **FDR** |
| Neurof_UnionInclusive | 2874 | 5.28E-04 | 0.03 | 2.30E-04 | 0.01 |
| Neurof_GoNervTransm | 716 | 1.33E-03 | 0.03 | 9.86E-04 | 0.02 |
| PSD_BayesGrant_fullset | 1407 | 1.29E-02 | 0.14 | 8.65E-03 | 0.10 |
| Neurof_GoSynaptic | 622 | 1.42E-02 | 0.14 | 9.87E-03 | 0.10 |
| Kirov_ARC | 28 | 1.03E-02 | 0.14 | 9.29E-03 | 0.10 |

*refer to Supplementary S2 for the full description of gene sets. FDR: false discovery rate


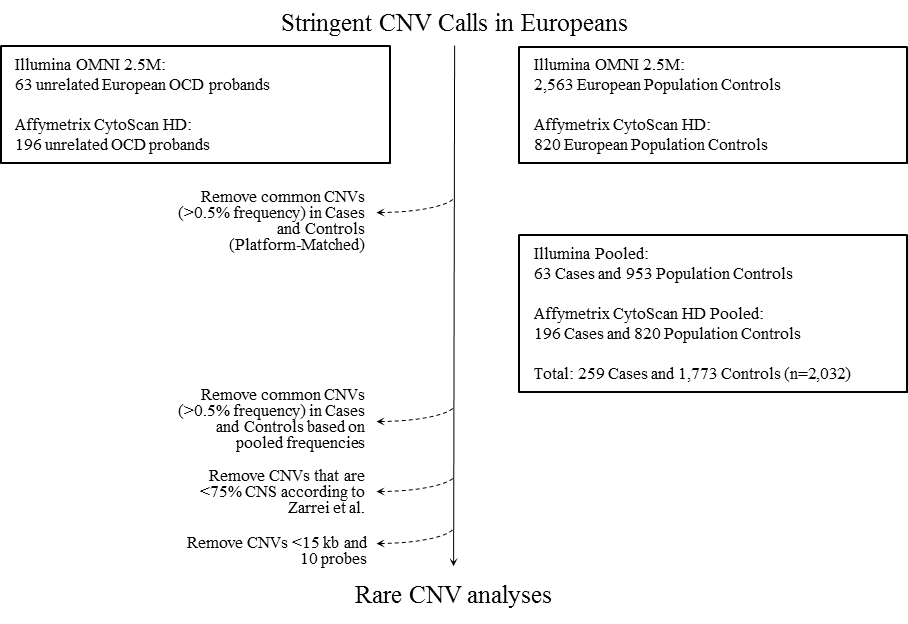


Supplementary Figure S1: Filtering for rare variants

This figure illustrates the pipeline that we used (and the number of cases and controls used at each step) to define our rare copy number variants.


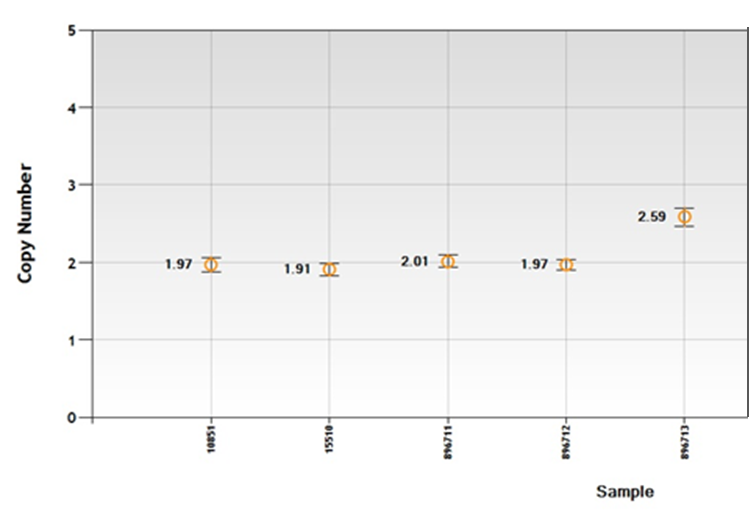


Supplementary Figure S2: Digital droplet PCR result for case D mosaic duplication at 10q11.22-q11.21 using Hs01220144_cn

This above figure shows the copy number validation assay performed for this mosaic duplication. Both controls (10851 and 15510) and parental samples (896711 and 896712) illustrate copy numbers of 2 while the copy number of the proband sample (896713) at this locus is 2.59.

**
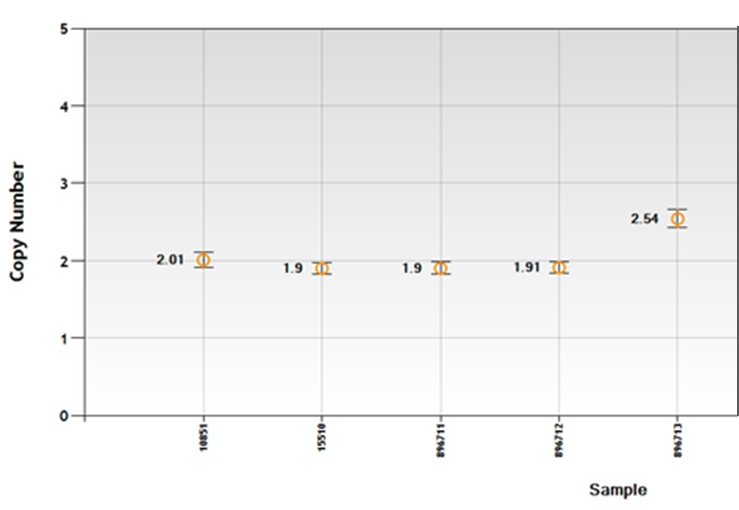
**

Supplementary Figure S3: Digital droplet PCR result for case D mosaic duplication at 10q11.22-q11.21 using Hs00847171_cn

This above figure shows the copy number validation assay performed for this mosaic duplication. Both controls (10851 and 15510) and parental samples (896711 and 896712) illustrate copy numbers of 2 while the copy number of the proband sample (896713) at this locus is 2.54.


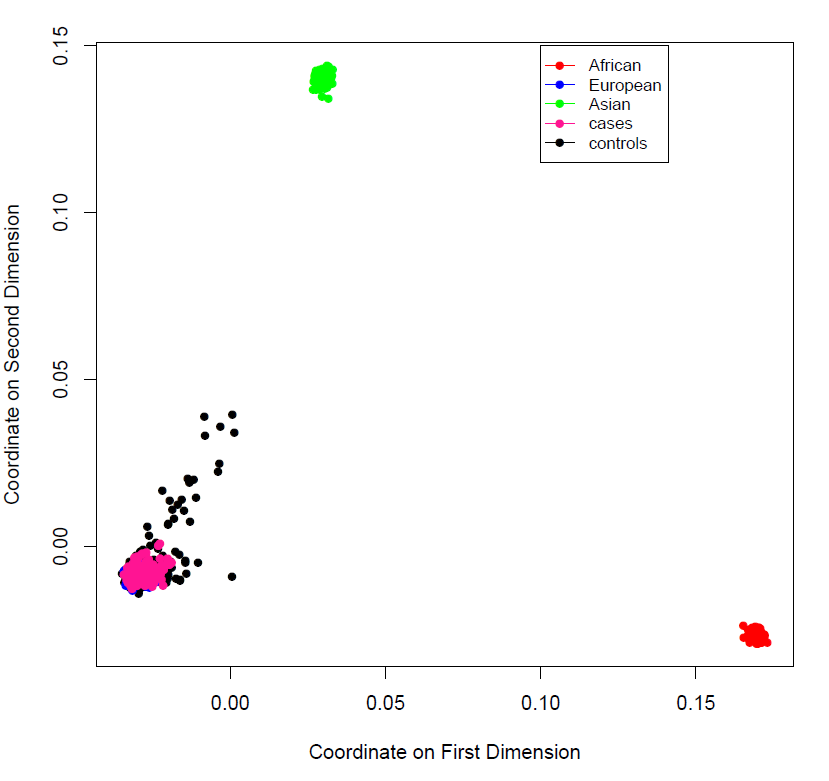


Supplementary Figure S4: Confirmation of European ancestry for cases and controls for use in gene set enrichment analysis

The two principle components from the multidimensional scaling analysis are plotted for those cases and controls determined to be European from our analysis.

**Table S1:** The list of rare copy number variants used in the current study [see online Table S1.xlsx]

**Table S2:** The list of gene sets used for the enrichment analysis in the current study [see online Table S2.xlsx]

**Table S5:** The results of enrichment analysis [see online Table S5.xlsx]

**ACKNOWLEDGEMENTS**

Control datasets were obtained, with permission for use, from the database of Genotypes and Phenotypes (dbGaP) (<http://www.ncbi.nlm.nih.gov/gap>; accession numbers phs000303.v1.p1 (Genetic Epidemiology of Refractive Error in the KORA Study) and phs000404.v1.p1 (Collaborative Genetic Study of Nicotine Dependence (COGEND) The Genetic Architecture of Smoking and Smoking Cessation)). The KORA dataset was obtained from the National Eye Institute (NEI) Refractive Error Collaboration (NEIREC) Database, with support from the NEI. COGEND samples were genotyped at the Center for Inherited Disease Research (CIDR), with support by 1 X01 HG005274-01. Assistance with genotype cleaning and study coordination was provided by the Gene Environment Association Studies (GENEVA) Coordinating Center (U01HG004446). Collection of COGEND datasets and samples was supported by COGEND P01 CA089392 and the University of Wisconsin Transdisciplinary Tobacco Use Research Center (P50 DA019706, P50 CA084724). The contents of this article are solely the responsibility of the authors and do not necessarily represent the official views of the NIH.

**References:**

1. Kaufman J, Birmaher B, Brent D, Rao U, Flynn C, Moreci P et al. Schedule for Affective Disorders and Schizophrenia for School-Age Children-Present and Lifetime Version (K-SADS-PL): initial reliability and validity data. Journal of the American Academy of Child and Adolescent Psychiatry. 1997;36(7):980-8.

2. Hanna GL. Schedule for Obsessive-Compulsive and Other Behavioral Syndromes. 2007.

3. Scahill L, Riddle MA, McSwiggin-Hardin M, Ort SI, King RA, Goodman WK et al. Children's Yale-Brown Obsessive Compulsive Scale: reliability and validity. Journal of the American Academy of Child and Adolescent Psychiatry. 1997;36(6):844-52.

4. R: A language and environment for statistical computing [database on the Internet]. R Foundation for Statistical Computing, Vienna, Austria 2016. Accessed:

5. Oskoui M, Gazzellone MJ, Thiruvahindrapuram B, Zarrei M, Andersen J, Wei J et al. Clinically relevant copy number variations detected in cerebral palsy. Nat Commun. 2015;6:7949. doi:10.1038/ncomms8949.

6. Gazzellone MJ, Zhou X, Lionel AC, Uddin M, Thiruvahindrapuram B, Liang S et al. Copy number variation in Han Chinese individuals with autism spectrum disorder. Journal of neurodevelopmental disorders. 2014;6(1):34. doi:10.1186/1866-1955-6-34.

7. Pinto D, Darvishi K, Shi X, Rajan D, Rigler D, Fitzgerald T et al. Comprehensive assessment of array-based platforms and calling algorithms for detection of copy number variants. Nature biotechnology. 2011;29(6):512-20. doi:10.1038/nbt.1852.

8. Darvishi K. Application of Nexus copy number software for CNV detection and analysis. Current protocols in human genetics / editorial board, Jonathan L Haines [et al]. 2010;Chapter 4:Unit 4 14 1-28. doi:10.1002/0471142905.hg0414s65.

9. Downey T. Analysis of a multifactor microarray study using Partek genomics solution. Methods in enzymology. 2006;411:256-70. doi:10.1016/S0076-6879(06)11013-7.

10. Wang K, Li M, Hadley D, Liu R, Glessner J, Grant SF et al. PennCNV: an integrated hidden Markov model designed for high-resolution copy number variation detection in whole-genome SNP genotyping data. Genome research. 2007;17(11):1665-74. doi:10.1101/gr.6861907.

11. Colella S, Yau C, Taylor JM, Mirza G, Butler H, Clouston P et al. QuantiSNP: an Objective Bayes Hidden-Markov Model to detect and accurately map copy number variation using SNP genotyping data. Nucleic acids research. 2007;35(6):2013-25. doi:10.1093/nar/gkm076.

12. Purcell S, Neale B, Todd-Brown K, Thomas L, Ferreira MA, Bender D et al. PLINK: a tool set for whole-genome association and population-based linkage analyses. American journal of human genetics. 2007;81(3):559-75. doi:10.1086/519795.

13. Pinto D, Delaby E, Merico D, Barbosa M, Merikangas A, Klei L et al. Convergence of genes and cellular pathways dysregulated in autism spectrum disorders. American journal of human genetics. 2014;94(5):677-94. doi:10.1016/j.ajhg.2014.03.018.

14. Zarrei M, MacDonald JR, Merico D, Scherer SW. A copy number variation map of the human genome. Nat Rev Genet. 2015;16(3):172-83. doi:10.1038/nrg3871.

15. Engchuan W, Dhindsa K, Lionel AC, Scherer SW, Chan JH, Merico D. Performance of case-control rare copy number variation annotation in classification of autism. BMC medical genomics. 2015;8(S7):1-10.

16. Lee C, Scherer SW. The clinical context of copy number variation in the human genome. Expert reviews in molecular medicine. 2010;12:e8. doi:10.1017/S1462399410001390.
